# A radiology-based differential diagnostic model for pelvic chondrosarcoma using random forest algorithms

**DOI:** 10.3389/fonc.2026.1818815

**Published:** 2026-05-01

**Authors:** Xiao Ma, Yutong Jiang, Nong Lin, Zhaoming Ye, Hengyuan Li

**Affiliations:** 1 Department of Orthopedic Surgery, The Second Affiliated Hospital, Zhejiang University School of Medicine, Hangzhou, Zhejiang, China; 2 Orthopedics Research Institute of Zhejiang University, Hangzhou, Zhejiang, China; 3 Key Laboratory of Motor System Disease Research and Precision Therapy of Zhejiang Province, Hangzhou, Zhejiang, China; 4 Clinical Research Center of Motor System Disease of Zhejiang Province, Hangzhou, China; 5 College of Information Science and Electronic Engineering, Zhejiang University, Hangzhou, China

**Keywords:** diagnostic model, differential diagnosis, pelvic chondrosarcoma, radiology, random forest

## Abstract

**Objective:**

To develop a non-invasive diagnostic method for pelvic chondrosarcoma using clinical and radiological features, aiming to improve early diagnostic accuracy and reduce reliance on invasive biopsies.

**Methods:**

Two hundred and thirty-eight patients (39.50% with pelvic chondrosarcoma; median age, 42.87 years; 53.78% male; all Chinese) were enrolled and assigned to training (n = 167) and testing (n = 71) cohorts. Seven clinical and radiological features were evaluated for their potential associations with pelvic chondrosarcoma diagnosis. A predictive model was developed using a random forest algorithm and validated in the testing cohort, with performance assessed by partial dependence plot analysis and additional accuracy metrics.

**Results:**

The likelihood of a positive diagnosis of pelvic chondrosarcoma was significantly associated with age around 50 years (p < 0.001), the presence of high-intensity signals on T2-weighted magnetic resonance imaging (p = 0.021), a ring-and-arc enhancement pattern on contrast-enhanced T1-weighted magnetic resonance imaging (p < 0.001), and intratumoral calcification (p < 0.001). Tumor location was also a critical determinant (p = 0.009), with acetabular tumors exhibiting higher diagnostic probability. Among all variables, the random forest model identified the ring-and-arc enhancement pattern and patient age at diagnosis as the two most influential predictors. The model achieved excellent diagnostic performance, with a sensitivity of 96.55%, specificity of 90.48%, and overall accuracy of 92.96%.

**Conclusion:**

Our random forest–based model provides a reliable and practical non-invasive diagnostic tool for pelvic chondrosarcoma, improving diagnostic accuracy while potentially reducing the reliance on invasive biopsy procedures and supporting clinical decision-making.

## Key points

- Question: Early diagnosis of pelvic chondrosarcoma remains challenging; biopsies carry risks of recurrence and sampling error, highlighting the need for reliable non-invasive diagnostic approaches.
- Findings: A random forest–based model using clinical and radiological features achieved 96.6% sensitivity, 90.5% specificity, and 93.0% accuracy in diagnosing pelvic chondrosarcoma.
- Clinical Relevance: This non-invasive predictive model enhances diagnostic accuracy for pelvic chondrosarcoma, potentially reducing unnecessary biopsies and supporting earlier, safer, and more precise clinical decision-making to improve patient management and prognosis.

## 1 Introduction

Chondrosarcoma is the prototypical form of chondrogenic tumors characterized by production of cartilaginous matrix (1). It constitutes approximately 20% of all primary bone malignancies (2), making it the second most prevalent type, following osteosarcoma (3). The median age at diagnosis is around 50 years (4, 5), with a slight male predominance (6). Conventional chondrosarcoma is typically managed through surgery; however, established treatment strategies are lacking for non-resectable, recurrent, and metastatic tumors due to their poor responses to chemo- and radiation therapy (7, 8).

Chondrosarcoma primarily manifests in the axial skeleton, with the pelvis being one of the most common sites, followed by proximal long bones (2, 9). Pelvic chondrosarcoma is typically associated with a higher recurrence rate (10) and poorer prognosis (11) compared to that in long bones. Due to the insidious onset of pain, diagnosis of pelvic chondrosarcoma often occurs after the tumor has reached a substantial size, which profoundly complicates resection efforts (12). Therefore, an early and accurate diagnosis is of pivotal importance in orthopedic clinical practice.

The role of presurgical biopsy for pelvic chondrosarcoma remains controversial (13). Although tissue biopsy is the most accurate method for differentiating chondrosarcoma pathologically, researchers also expressed concerns regarding its reliability (14). This challenge might arise from the tumor’s heterogeneity and the complex anatomical structure of the pelvis, which complicates the identification of an optimal biopsy site (15). Moreover, biopsies pose a risk of local recurrence, due to potential dissemination of tumor cells along the biopsy tract (16), but the definitive impact of biopsies on local recurrence continues to be debated (17).

To date, some studies have indicated that diagnosing low-grade chondral lesions (including enchondroma and low-grade chondrosarcoma) solely through imaging, without the need for presurgical biopsy, is a safe approach (18, 19). The characteristic radiological features of pelvic chondrosarcoma assessed in these studies included matrix calcification, cortical destruction, trabecular penetration and extra-skeletal soft tissue mass (20). Additionally, due to its cartilaginous composition, chondrosarcoma typically displays as hyaline cartilage nodules with lobular architecture and high water content, effectively visualized through MRI (21). These distinctive features facilitate the specific diagnosis of chondrosarcoma, thereby holding substantial clinical significance.

Therefore, in the present study, we incorporated seven significant clinical and radiological features of pelvic chondrosarcoma previously documented in the literature. We conducted a comprehensive analysis of the data and developed a diagnostic model based on random forest algorithms, utilizing a relatively large patient cohort despite the disease’s rarity. The model demonstrated a satisfactory predictive value with an accuracy rate of over 90%.

## 2 Materials and methods

### 2.1 Patient selection

**Figure 1:**
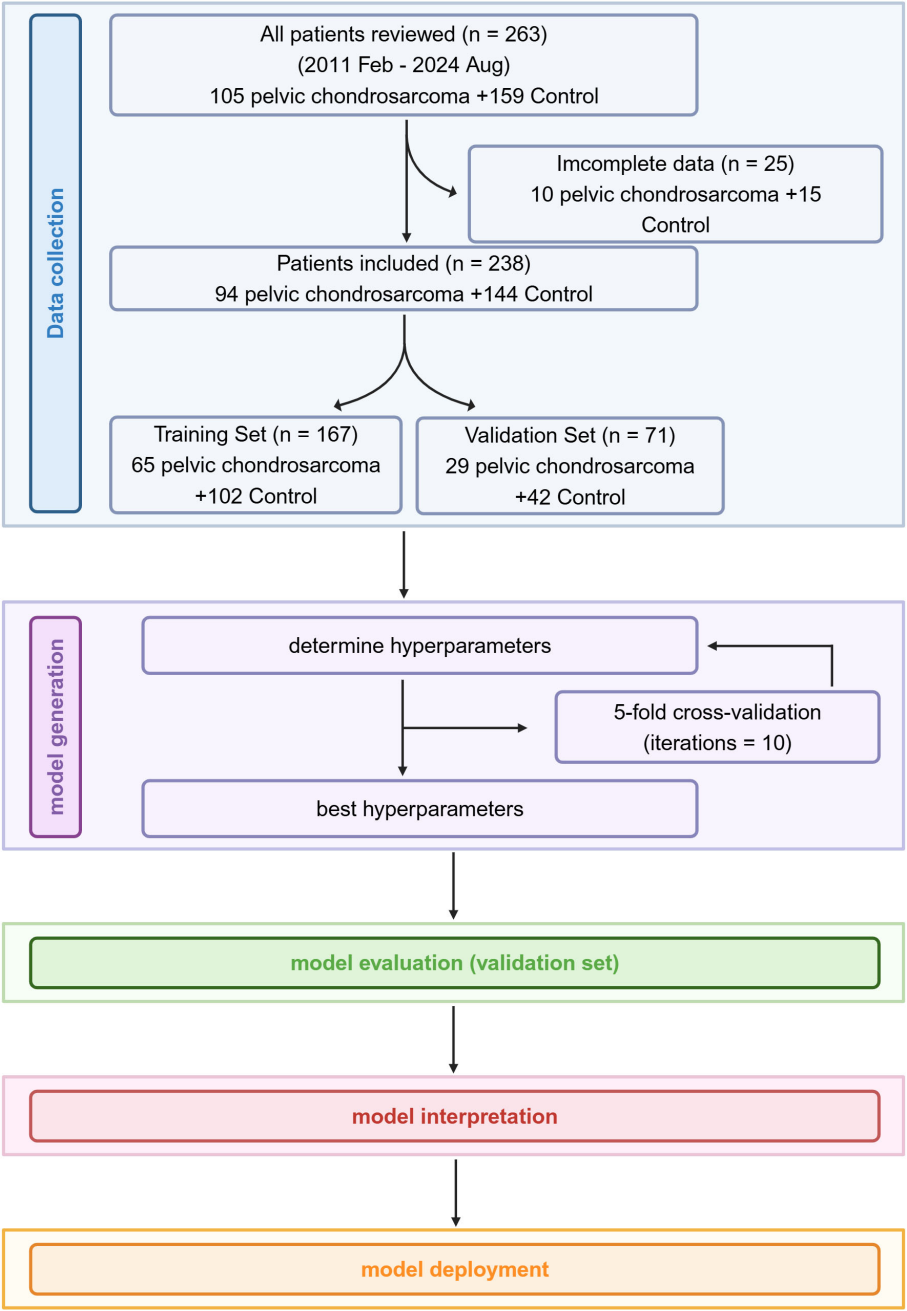
Flow diagram of the study. Created using biorender.com.

Approval of the study was granted by the hospital’s Medical Ethical Committee (Number I20241271). The requirement for written informed consent was waived by the institutional review board due to the retrospective nature of the study and the use of anonymized data. The present retrospective study included a total of 104 patients diagnosed with pelvic chondrosarcoma treated at the Orthopedic Department of the Second Affiliated Hospital of Zhejiang University School of Medicine, along with 159 patients in the control group, between February 2011 and August 2024 (Research workflow showed in **Figure 1**). The inclusion criteria were: (1) tumor located in the pelvis; (2) histopathological confirmation of chondrosarcoma or other conditions via surgically obtained specimens. Exclusion criteria included: (1) patients who had undergone tumor resection prior to admission; (2) incomplete data; (3) patients primarily treated at other centers. Ultimately, 94 patients in the chondrosarcoma group and 144 in the control group met the inclusion criteria. All patients underwent preoperative CT scans and contrast-enhanced MRI examinations, and surgical specimens were histologically evaluated to establish the final diagnosis. The control group consisted of patients with other bone malignancies or benign conditions (**Table 1**). The study population comprised 128 men and 110 women, with a mean age of 42.87 years (range: 6–78 years); all patients enrolled are of Chinese origin. The 238 patients were randomly divided into a training cohort (n =167) and a testing cohort (n = 71) in a 70:30 ratio. Pelvic chondrosarcoma patients constituted 39.50% of the total, with 38.92% in the training set and 40.85% in the testing set. Age and sex distributions were consistent across both cohorts (**Table 2**).

**Table 1:** Pathological diagnosis of patients in control group.

Pathological diagnosis	Number of patients
Osteosarcoma	24
Giant cell tumor of bone	20
Langerhans cell histiocytosis (LCH)	15
Bone cyst	8
Fibrous dysplasia	8
Undifferentiated sarcoma	6
Metastatic thyroid cancer	6
Aneurysmal bone cyst	5
Enchondroma	5
Ewing’s sarcoma	5
Non-ossifying fibroma	3
Smooth muscle sarcoma	3
Chondroblastoma	3
Metastatic colorectal cancer	3
Other primary benign space-occupying lesions	13
Other primary malignant tumors	11
Other metastatic malignant tumors	6
Total	144

**Table 2:** Summary of patient data enrolled in the study.

Parameters	Training cohort (n = 167)	Validation cohort (n = 71)	Total (n = 238)
Age	42.95 ± 17.55	42.69 ± 19.93	42.87 ± 18.25
Gender			
Male	89 (53.29%)	39 (54.93%)	128 (53.78%)
Female	78 (46.71%)	32 (45.07%)	110 (46.22%)
Diagnosis			
Chondrosarcoma	65 (38.92%)	29 (40.85%)	94 (39.50%)
Non-chondrosarcoma	102 (61.08%)	42 (59.15%)	144 (60.50%)
Location			
Ilium	83 (49.70%)	36 (50.70%)	119 (50.00%)
Acetabulum	47 (28.14%)	16 (22.54%)	63 (26.47%)
Ischium and pubis	37 (22.16%)	19 (26.76%)	56 (23.53%)
Soft tissue mass			
Yes	123 (73.65%)	56 (78.87%)	179 (75.21%)
No	44 (26.35%)	15 (21.13%)	59 (24.79%)
High signal intensity on T2-weighted MRI			
Yes	156 (93.41%)	66 (92.96%)	222 (93.28%)
No	11 (6.59%)	5 (7.04%)	16 (6.72%)
Ring-and-arc enhancement pattern on enhanced T1-weighted MRI			
Yes	65 (38.92%)	28 (39.44%)	93 (39.08%)
No	102 (61.08%)	43 (60.56%)	145 (60.92%)
Intratumoral calcification			
Yes	89 (53.29%)	38 (53.52%)	127 (53.36%)
No	78 (46.71%)	33 (46.48%)	111 (46.64%)

### 2.2 Radiological characteristics of the tumor

The results were reviewed by at least two experienced radiologists or orthopedic surgeons to ensure their reliability. In cases of disagreement, a consensus was reached through joint discussion. To further assess the reliability of feature evaluation, inter-observer agreement was quantified using Cohen’s kappa coefficients. X-rays and PET-CT were utilized as supplementary assessments for certain patients. The criteria for each radiological characteristic are described as follows (the results for each feature were recorded as either “yes” or “no”, except for tumor location):

Location: In the present study, the pelvis is categorized into four regions (22): ilium (location 1), acetabulum (location 2), ischium and pubis (location 3), and sacrum (location 4). The tumor’s location was identified based on its origin; if the origin is not clear, the site with the majority of the tumor mass was selected. It should be stated that no patients enrolled in this study manifested a sacrum tumor due to its extreme rarity.
Soft tissue mass: The tumor manifests as an extra-skeletal soft tissue mass, exhibiting cortical penetration or destruction (21).
High signal intensity on T2-weighted MRI: The tumor features very high intensity in non-mineralized or calcified regions when evaluated with T2-weighted MRI, as the cartilage is a hydrophilic tissue with high water content. Areas with low signal intensity typically correspond to sites of chondroid mineralization observed on CT scans (23).
Ring-and-arc enhancement pattern on contrast-enhanced T1-weighted MRI (Gd): The tumor displays a lobulated structure with septal and peripheral rim-like enhancement when evaluated with enhanced T1-weighted MRI, reflecting fibrovascular septation between the lobules of hyaline cartilage (23, 24).
Intratumoral calcification: The tumor features intratumoral mineralization or calcification, especially when evaluated with CT scan (21, 25).

The inter-observer agreement for the five radiological features demonstrated excellent reproducibility, with Cohen’s kappa values of 0.953 for location, 0.889 for soft tissue mass, 0.902 for high signal intensity on T2-weighted MRI, 0.956 for ring-and-arc enhancement on contrast-enhanced T1-weighted MRI, and 0.949 for intratumoral calcification.

### 2.3 Random forest decision

**Figure 2:**
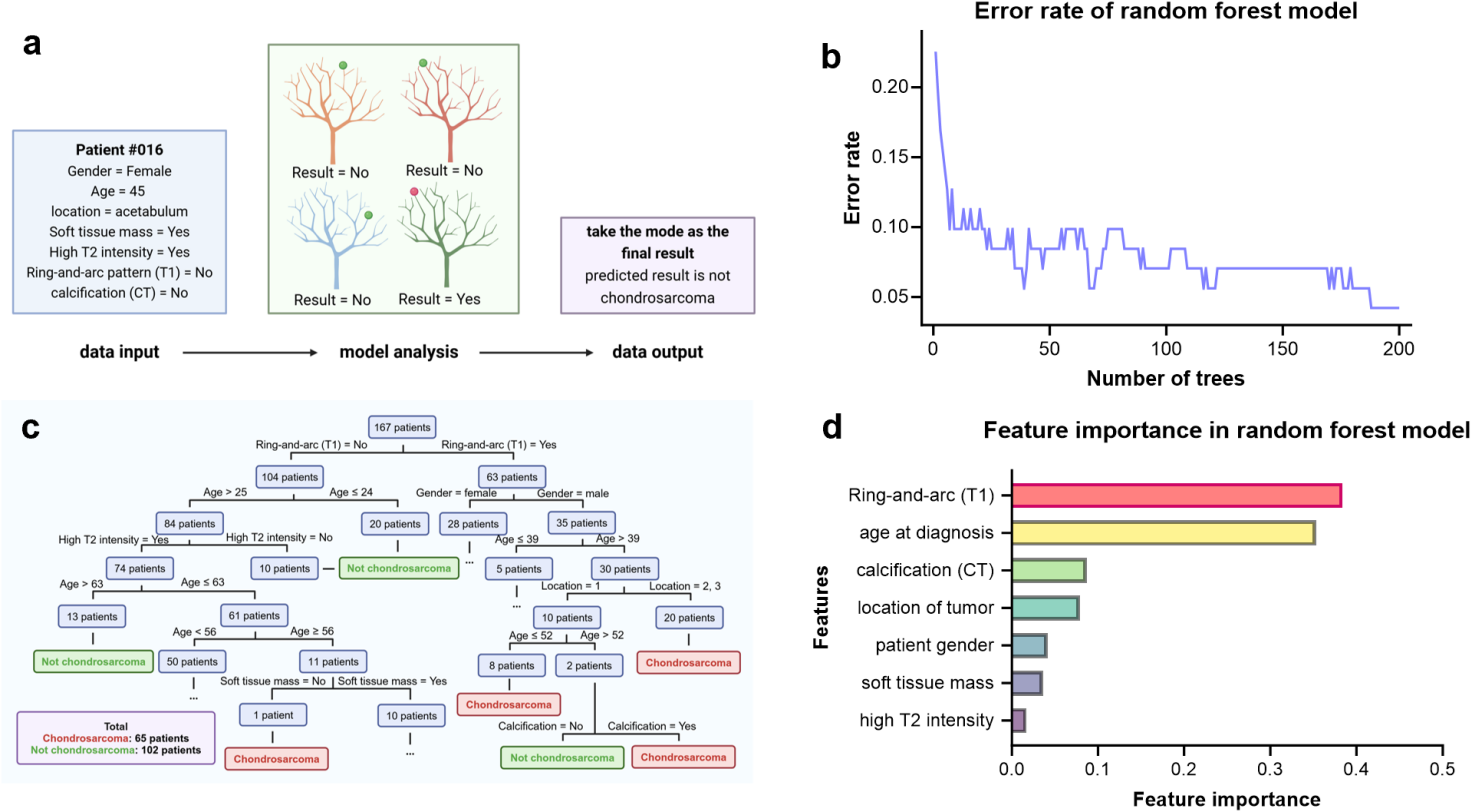
Construction of the random forest model. **(a)** Model diagram illustrating how random forest model works. **(b)** The relationship between the error rate of the model and the number of trees growing. **(c)** Schematic diagram displaying part of a decision tree. **(d)** Feature importance of the seven selected features.

Random forest is an ensemble supervised machine learning strategy that utilizes multiple decision trees, each constructed from randomly selected sub-datasets. This decision method enhances both accuracy and stability by aggregating the predictions of various trees, which helps to mitigate overfitting and improve generalization to new data. The final classification result is determined by taking the mode of the results predicted by all individual trees, ensuring that the most common outcome is selected (26, 27) (**Figure 2a**).

In this study, key hyperparameters were carefully chosen to optimize performance. **Figure 2b** illustrates the error rate as a function of the number of decision trees, ranging from 1 to 200. The error rate is defined as the proportion of incorrect predictions on the testing set. As the number of trees increases, the error rate generally decreases, indicating improved model performance. However, this improvement comes at the cost of longer training times and higher computational demands. We ultimately selected 100 decision trees as the optimal configuration, striking a balance between minimizing the error rate and maintaining training efficiency. In addition, the trees were allowed to grow without depth restrictions. We specified that the minimum number of samples required to split a node was two, with at least one sample in each leaf. Furthermore, each tree used the square root of the total number of features. This combination of parameters was designed to achieve an optimal balance between model complexity and predictive accuracy.

### 2.4 PDP analysis

In this study, partial dependence analysis was employed to specifically interpret the influence of each selected feature on the predicted diagnosis (28, 29), by calculating the average percentage of predicted positive chondrosarcoma diagnoses, while marginalizing over the other features. Partial dependence was defined as:


$${\hat{f}}_{x_{S}}(x_{S})=E_{x_{C}}[\hat{f}(x_{S},x_{C})]=\int \hat{f}(x_{S},x_{C})dP(x_{C})$$

where $x_{S}$ denotes a single feature of interest (in this study, each one of the seven parameters), $x_{C}$ represents the set of other features, $\hat{f}$ refers to the trained random forest model, and E indicates the expectation over the distribution of $x_{C}$. This analytical approach quantifies the unique contribution of each feature to the predictive model, enhancing the interpretability of the diagnostic process and supporting more informed clinical decision-making.

### 2.5 Statistical analyses

Statistical analyses were conducted using software IBM SPSS Statistics version 27.0.1. Descriptive statistics were computed for both continuous and categorical variables. Univariate logistic regression was utilized to evaluate the association between age and positive diagnoses of pelvic chondrosarcoma, with odds ratio (OR) and 95% confidence interval (CI) calculated. Additionally, the Chi-square test was employed to assess other parameters between the pelvic chondrosarcoma and control groups, with Fisher’s exact test applied for expected frequencies below 5. A p-value < 0.05 was considered statistically significant for all analyses.

## 3 Results

### 3.1 Correlation between selected features and pathological diagnosis

We first examined the association between selected features and the positive pathological diagnosis of pelvic chondrosarcoma. The average age of patients with pelvic chondrosarcoma was 49.37 ± 13.14 years old, aligning with previous research that indicated a peak incidence around age 50 (5, 6, 30). Considering the non-linear relationship between patient age and chondrosarcoma incidence, the difference from 50 years was selected as the analytical indicator. Logistic regression analysis revealed a significant link between proximity to age 50 and the positive diagnosis (p < 0.001, OR = 0.934 [0.902 – 0.967]). Other parameters were analyzed using the Chi-square test, among which the presence of high signal intensity on T2-weighted MRI (p = 0.021), a ring-and-arc enhancement pattern on enhanced T1-weighted MRI (p < 0.001), and intratumoral calcification (p < 0.001) were significantly related to the diagnosis of pelvic chondrosarcoma (**Table 3**). Additionally, tumor location also significantly impacted diagnostic outcomes (p = 0.009), with acetabulum tumors more likely to yield a positive diagnosis (location 1 vs. 2, p = 0.006, OR = 0.357; location 2 vs. 3, p = 0.011, OR = 3.191). However, gender and the presence of soft tissue mass did not show a significant correlation with the positive diagnosis.

**Table 3:** Analysis of parameters using training cohort.

Parameters	Chondrosarcoma (n = 65)	Non-chondrosarcoma (n = 102)	OR	p value
Age	49.37 ± 13.14	38.85 ± 18.80	/	/
Gender			1.148	0.665
Male	36 (55.38%)	53 (51.96%)		
Female	29 (44.62%)	49 (48.04%)		
Location			/	0.009
Ilium (zone 1)	27 (41.54%)	56 (54.90%)		
Acetabulum (zone 2)	27 (41.54%)	20 (19.61%)		
Ischium and pubis (zone 3)	11 (16.92%)	26 (25.49%)		
Soft tissue mass			2.019	0.065
Yes	53 (81.54%)	70 (68.63%)		
No	12 (18.46%)	32 (31.37%)		
High signal intensity on T2-weighted MRI			6.957	0.021
Yes	64 (98.46%)	92 (90.20%)		
No	1 (1.54%)	10 (9.80%)		
Ring-and-arc enhancement pattern on enhanced T1-weighted MRI			33.125	<0.001
Yes	53 (81.54%)	12 (11.76%)		
No	12 (18.46%)	90 (88.24%)		
Intratumoral calcification			4.201	<0.001
Yes	48 (73.85%)	41 (40.20%)		
No	17 (26.15%)	61 (59.80%)		

### 3.2 Establishment of the random forest prediction model

In the current study, the number of decision trees was set to 100 in the random forest model. **Figure 2c** illustrates an example of a decision tree. Among all the seven parameters, the ring-and-arc enhancement pattern on T1-weighted MRI and age at diagnosis exhibited the highest diagnostic values, as depicted in the feature importance plot (**Figure 2d**). The ring-and-arc contrast enhancement pattern is a typical feature of chondrosarcoma, reflecting the capillary structures in the septa and perichondrium that facilitate tumor blood supply (31). In addition, intratumoral calcification and tumor location followed in importance, highlighting the osteogenic characteristics of chondrogenic tumors and the influence of location. Comparatively, the last three features—gender, soft tissue mass, and high signal intensity on T2-weighted MRI—contributed less to a positive diagnosis.

### 3.3 Model performance

**Figure 3:**
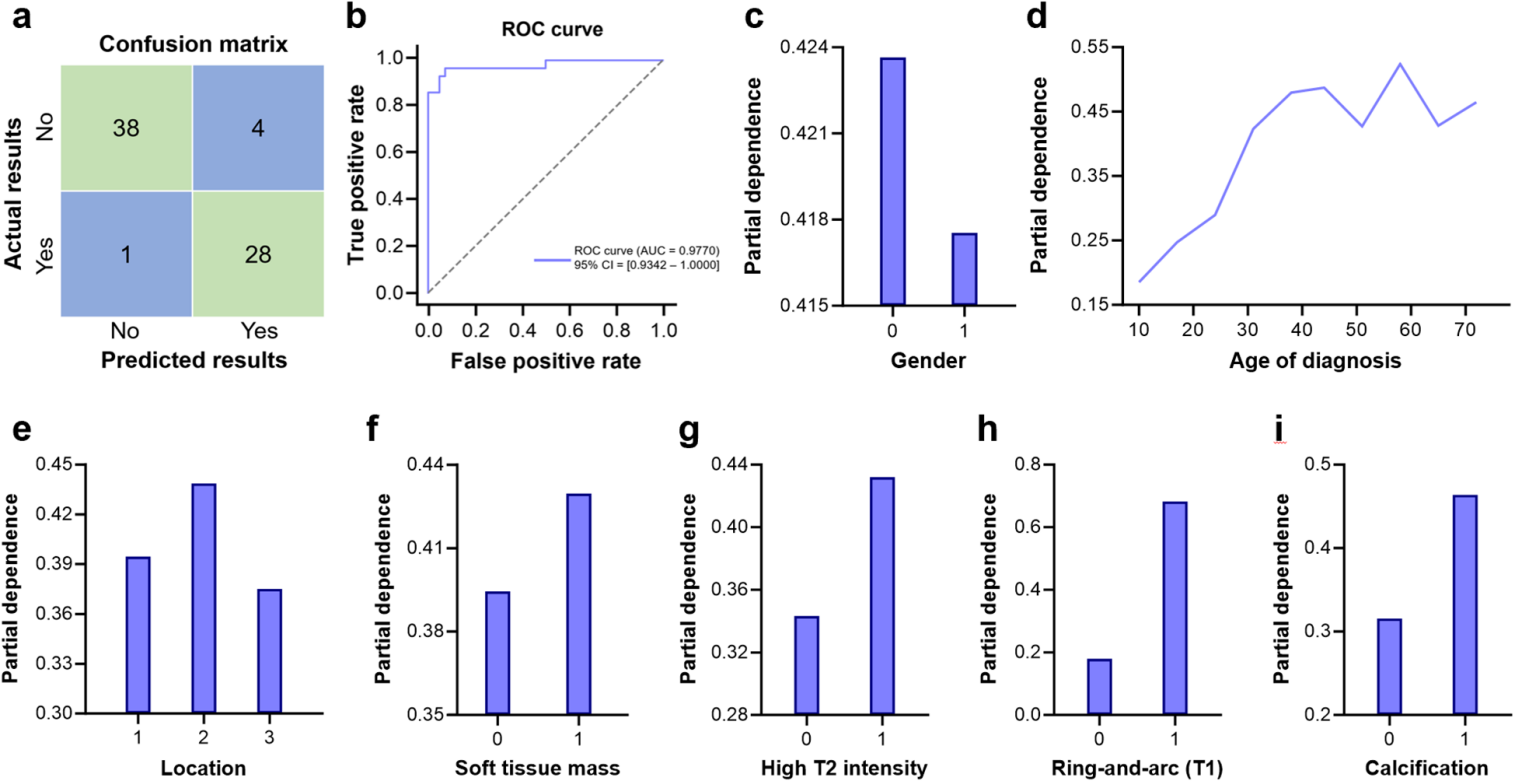
The assessment of the diagnostic model and each selected feature. **(a)** Confusion matrix plot illustrating the actual and predicted results in the validation set. **(b)** ROC curve of the model based on the validation set. **(c–i)** Partial dependence plots of gender, age of diagnosis, tumor location, the presence of soft tissue mass, high intensity signals on T2-weighted MRI, a ring-and-arc enhancement pattern on T1-weighted MRI, and calcification. X-axis represents the values of the features, and Y-axis indicates their predictive effectiveness.

Next, the validation set was employed to test the predictive efficacy of the model, and the confusion matrix plot illustrated the actual and predicted results (**Figure 3a**). The sensitivity, specificity, negative predictive value, positive predictive value, and the correctness of the overall predictive model were 96.55%, 90.48%, 97.44%, 87.50% and 92.96%, respectively. The Receiver Operating Characteristic (ROC) curve was plotted, and they are under the curve (AUC) was 0.9770 [0.9342 – 1.0000] (**Figure 3b**). Overall, our random forest–based predictive diagnostic model demonstrated satisfactory accuracy.

### 3.4 Interpretation of the features

**Figure 4:**
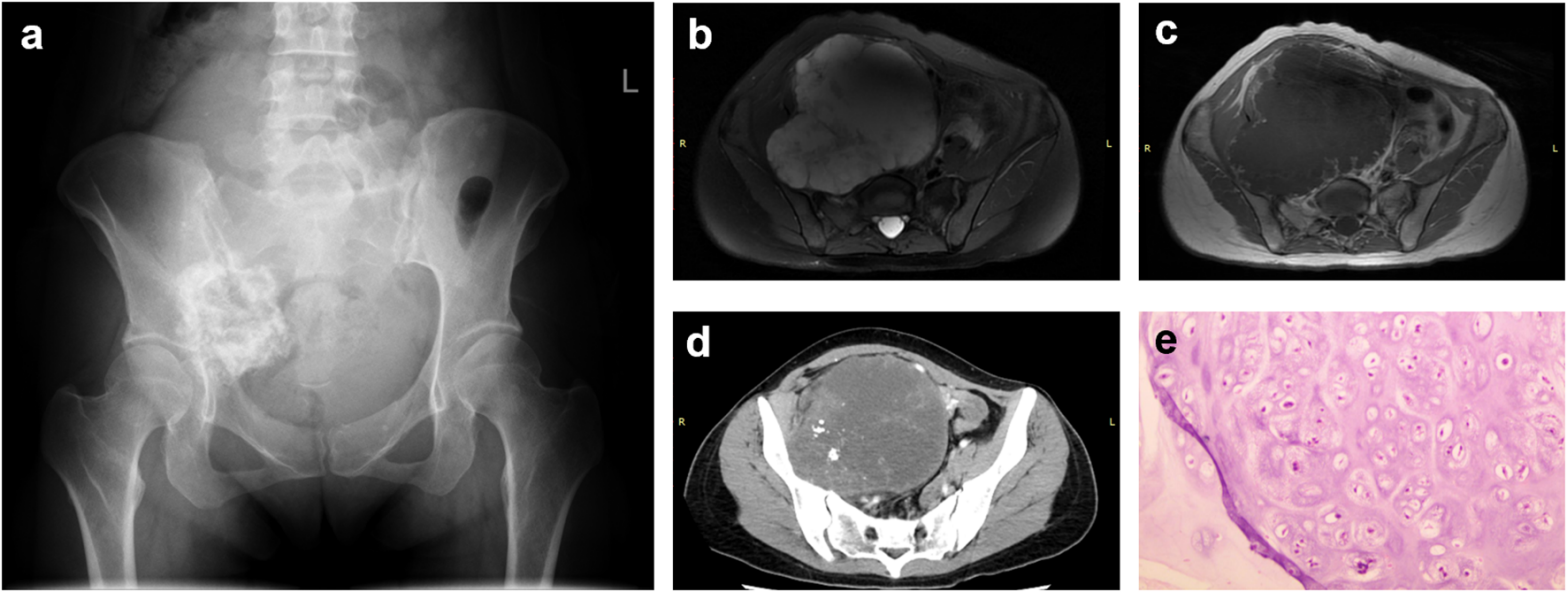
Case 1. A 33-year-old woman presented with a mass-like lesion exhibiting increased density, unclear borders, and bone destruction in the right iliac bone on X-ray **(a)**. MRI showed a mass with high intensity on T2-weighted (fat suppression) imaging **(b)** and a rim-and-arc pattern on enhanced T1-weighted imaging, indicating tumor blood supply **(c)**. CT scan revealed a mass with scattered high-density lesions corresponding to calcification sites **(d)**. Both the actual and predicted pathological diagnoses **(e)** were pelvic chondrosarcoma.

**Figure 5:**
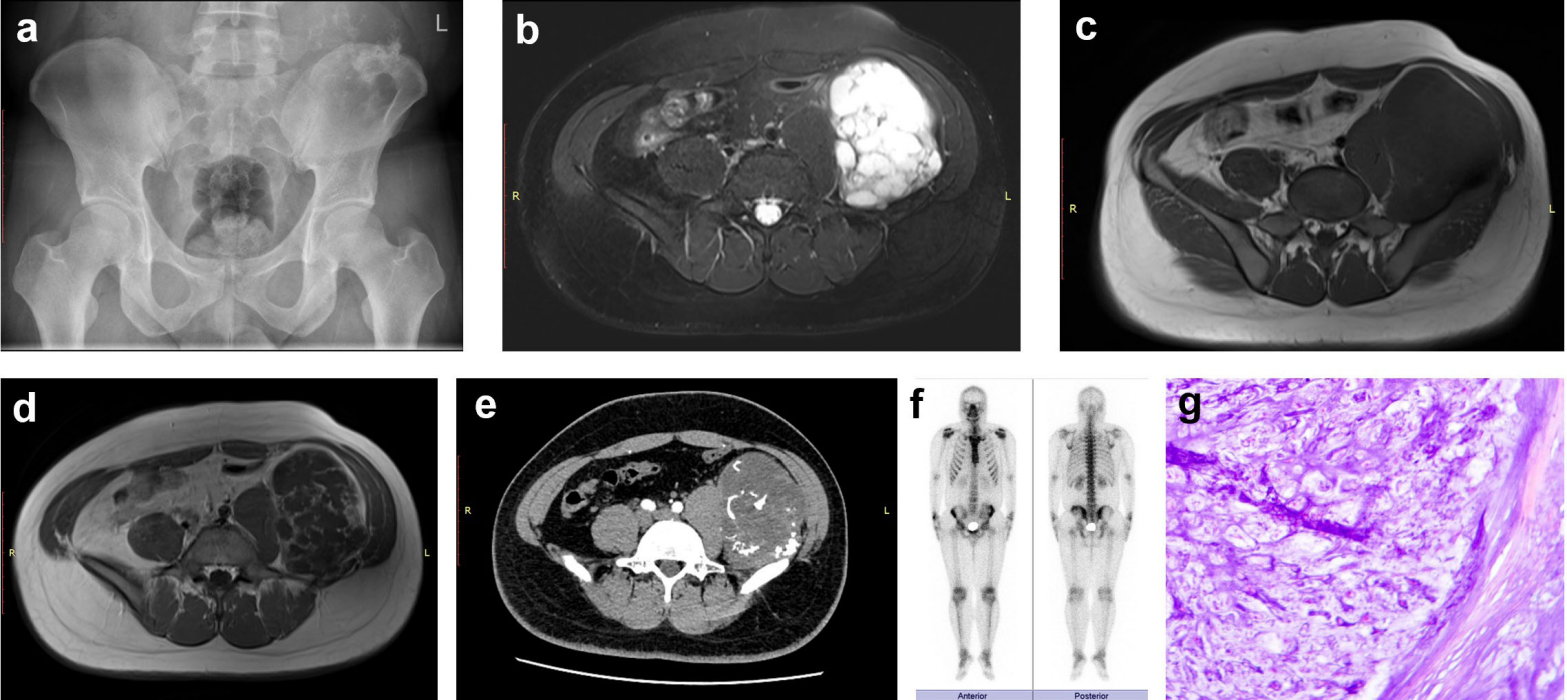
Case 2. A 25-year-old man showed increased bone density in the left iliac crest of the pelvis, with irregular calcification and slight swelling in the surrounding areas, as illustrated on X-ray **(a)**. MRI findings showed a water-rich mass with very high intensity on T2-weighted (fat suppression) imaging **(b)** and low intensity on T1-weighted imaging **(c)**, whereas enhanced MRI displayed a characteristic rim-and-arc pattern on T1-weighted images, with clear enhancement of the fibrovascular septa and tumor envelope **(d)**. CT scan presented intratumoral ossification **(e)**, and a whole-body bone scan indicated a mildly concentrated radioactive distribution in the left iliac crest **(f)**. Both the actual and predicted pathological diagnoses **(g)** were pelvic chondrosarcoma.

**Figure 6:**
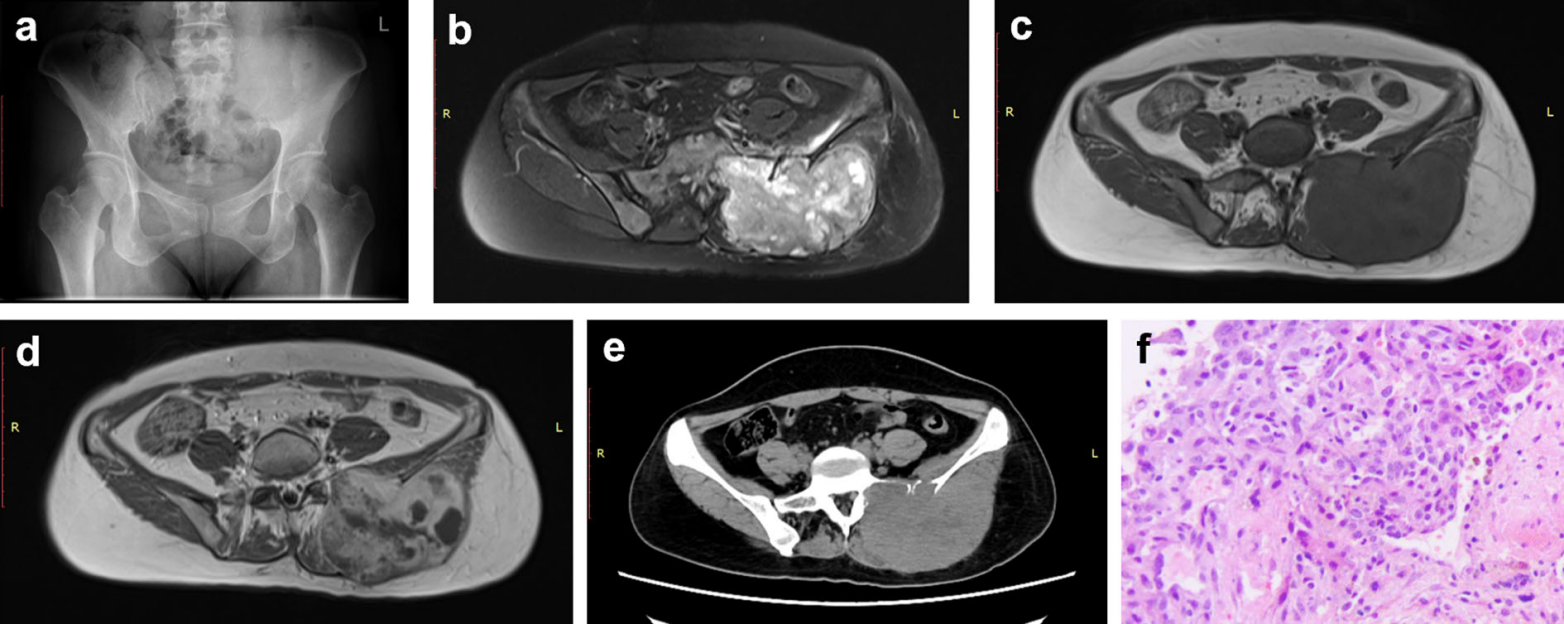
Case 3. A 47-year-old man was diagnosed with the giant cell tumor of bone. X-ray revealed an unclear sacroiliac joint space **(a)**. MRI showed heterogenous high intensity on T2-weighted (fat suppression) images **(b)** and low intensity in T1-weighted images **(c)**. The T1-weighted imaging also displayed a mixed enhancement pattern **(d)**. No calcification was observed on the CT scan **(e)**. Neither the actual nor the predicted pathological diagnoses **(f)** were pelvic chondrosarcoma.

To better elucidate the diagnostic features, the partial dependence analysis was conducted for each individual feature, revealing how their distributions influenced the diagnostic outcome. The results were visualized, with the X-axis representing the values of the features and the Y-axis indicating their predictive effectiveness. As anticipated, the male gender, soft tissue mass, high signal intensity on T2-weighted MRI, ring-and-arc enhancement pattern on T1-weighted MRI, and intratumoral calcification, were associated with an increased likelihood of pelvic chondrosarcoma (**Figures 3c, f–i**). Notably, for adolescents and young adults, the propensity of a positive diagnosis escalated drastically with age, while for individuals over 40, the age of onset exhibited variable diagnostic significance (**Figure 3d**). Furthermore, tumor location at the acetabulum was linked to a higher likelihood of pelvic chondrosarcoma (**Figure 3d**), corroborating our previous findings from the Chi-square test. Typical cases are illustrated in **Figures 4**–**6**. Overall, our random forest–based model demonstrated a commendable diagnostic performance for pelvic chondrosarcoma.

## 4 Discussion

Chondrosarcomas are malignant bone tumors that can either arise *de novo* or develop from preexisting cartilaginous lesions like osteochondromas and enchondromas. Approximately 30% of all chondrosarcoma cases occur in the pelvic region (32). The surgical excision of pelvic chondrosarcomas presents a unique challenge due to the complexity of reconstructing weight-bearing anatomical structures (33), underscoring the importance of early diagnosis and intervention. However, pelvic chondrosarcomas often present with nonspecific signs such as pain, a palpable mass and restricted motion; some cases may even be asymptomatic (34). Currently, while biopsy is considered the “gold standard” for diagnosis, it carries risks such as tumor seeding, missed diagnosis, technical difficulty, and potential complications for subsequent surgery. Therefore, developing accurate, non-invasive diagnostic methods is crucial for timely diagnosis and improving patient outcomes.

Previous studies have suggested that, in selected cases, imaging-based diagnoses for low-grade chondrosarcoma can be reliable without presurgical biopsy. For example, M.T. Brown et al. and Onur Berber et al. argued that needle biopsy is unnecessary when radiological features suggest benign or mildly malignant conditions, such as enchondroma or low-grade chondrosarcoma (18, 19). However, in routine clinical practice, histopathological confirmation remains essential and cannot be fully replaced. In this context, our model can be considered as a non-invasive adjunct tool to support pre-biopsy risk stratification, particularly in deep pelvic locations where image-guided percutaneous biopsy may be technically challenging and associated with potential risks such as tumor seeding along the biopsy tract. Furthermore, radiomics has been employed to distinguish chondrosarcoma from osteosarcoma (35) and atypical cartilaginous tumors (36), assess the histopathological grading of chondrosarcoma (37), and predict patient outcomes (38). These studies underscored the feasibility of using clinical and radiological characteristics for diagnosis. However, a standardized evaluation approach remains lacking, particularly for pelvic chondrosarcoma, as this region has never been the focus of previous studies.

Therefore, in this study, we focused on seven clinically and radiologically significant features of pelvic chondrosarcoma, as identified from previous reports using CT and contrast-enhanced MRI. Remarkably, a piece of code was provided for immediate use. To the best of our knowledge, this is the first systematic analysis to integrate these characteristics of pelvic chondrosarcoma and develop an easy-to-use prognostic model. The model trained on patient data collected over 13.5 years achieved over 90% predictive accuracy. Importantly, the primary aim of our model was not to identify novel imaging biomarkers, but to quantitatively integrate these known semantic features into a standardized and reproducible decision-supporting framework. The selected clinical parameters—age and gender—are easily obtainable; the imaging features, including tumor location, soft tissue mass, high intensity on T2-weighted MRI, ring-and-arc enhancement pattern on contrast-enhanced T1-weighted MRI, and intratumoral calcification, can all be assessed through standard radiological examinations. Our results indicated that the ring-and-arc enhancement pattern and patient age were the two most significant factors for the final diagnosis. This finding aligns with previously reported characteristic septal and nodular enhancement patterns of chondrosarcoma (23), while emphasizing the importance of age in the diagnostic process—a factor that had not been highlighted previously. Further analysis through partial dependence plots revealed that younger patients are less likely to have chondrosarcoma despite its peak incidence around age 50. Conversely, soft tissue mass—a typical feature of chondrosarcoma—demonstrated less significant in our diagnostic model, likely due to its common presence in other pelvic malignancies. Additionally, our study pointed out that the location of tumor within the pelvis plays a significant role in the likelihood of chondrosarcoma, with the acetabulum being the most common site. Furthermore, the association between intratumoral calcification and pelvic chondrosarcoma was weaker than expected. This may be attributed to the presence of benign cartilaginous lesions which also exhibit chondrogenic features, as well as the observation that calcification tends to decrease as the degree of malignancy and dedifferentiation increase (39).

Despite its clinical relevance, our study has several limitations. First, the rarity of pelvic chondrosarcoma limits the sample size of patients included. In addition, this was a single-center study without an independent external validation cohort, which may restrict the generalizability of our findings. Future multi-center studies with larger and more diverse populations are warranted to further validate the model. Second, the control group in our study was relatively heterogeneous, including multiple non-cartilaginous pathologies that are generally less challenging to distinguish from chondrosarcoma compared to cartilaginous lesions such as enchondroma. This imbalance in diagnostic difficulty may have led to an overestimation of the model’s performance in real-world clinical scenarios. Third, we focused on only seven key diagnostic features selected based on existing literature and personal clinical experience. Although these features are clinically interpretable, other potential features, such as serum calcium levels, may also play a part in chondrosarcoma diagnosis, and could be explored in future research. Fourth, the assessment of certain radiological characteristics inherently involves a degree of subjectivity and depends on the expertise of the interpreting radiologists. Although inter-observer agreement was acceptable in our study, this subjectivity remains a limitation of semantic feature-based models. Finally, while semantic features offer strong clinical interpretability, radiomics-based approaches may provide more objective and reproducible imaging biomarkers by extracting high-dimensional quantitative features (40–43). For instance, a machine learning model based on radiomic features extracted from unenhanced T1-weighted MRI showed satisfactory diagnostic performance for classification of low- to high-grade cartilaginous bone tumors, comparable to that of experienced musculoskeletal radiologists (42). However, radiomic feature stability is also influenced by inter-observer variability and segmentation approaches (43). Compared with radiomics-based models, which often rely on large numbers of abstract and less interpretable features, semantic feature–based models offer greater clinical interpretability and ease of implementation in routine practice, albeit potentially at the cost of reduced feature complexity. Future work should explore the integration of semantic and radiomic features to develop hybrid models that combine interpretability with improved predictive performance and generalizability.

## 5 Conclusion

In this study, we established a diagnostic model for pelvic chondrosarcoma using random forest algorithms, based on clinical and radiological parameters, which showed high accuracy and potential for clinical application. The two most influential factors were the ring-and-arc enhancement pattern on T1-weighted MRI and the patient’s age at onset. This model offers a straightforward, reliable and easy-to-use diagnostic approach to aid clinical decision-making and, if further validated, may serve as a non-invasive adjunct tool for preoperative assessment, particularly in cases where biopsy is technically challenging.

## Data Availability

The raw data supporting the conclusions of this article will be made available by the authors, without undue reservation.
